# Anti-inflammatory effect of hesperidin enhances chondrogenesis of human mesenchymal stem cells for cartilage tissue repair

**DOI:** 10.1186/s12950-018-0190-y

**Published:** 2018-07-20

**Authors:** Shipeng Xiao, Wenguang Liu, Jianqiang Bi, Shenghou Liu, Heng Zhao, Ningji Gong, Deguo Xing, Hongwei Gao, Mingzhi Gong

**Affiliations:** 1 Department of Joint Surgery, The Second Hospital of Shandong University, Jinan, 250033 Shandong China; 2 Key Laboratory for Liquid-Solid Structural Evolution and Processing of Materials, Ministry of Education, Shandong University, Jinan, 250061 Shandong China; 3 Department of emergency, The Second Hospital of Shandong University, Jinan, 250033 Shandong China; 4 Department of Trauma and Orthopedics, The Second Hospital of Shandong University, Jinan, 250033 Shandong China

**Keywords:** Articular cartilage, Hesperidin, Mesenchymal stem cells, Chondrogenesis, Inflammation

## Abstract

**Background:**

Articular cartilage diseases are considered a major health problem, and tissue engineering using human mesenchymal stem cells (MSCs) have been shown as a promising solution for cartilage tissue repair. Hesperidin is a flavonoid extract from citrus fruits with anti-inflammatory properties. We aimed to investigate the effect of hesperidin on MSCs for cartilage tissue repair. MSCs were treated by hesperidin, and colony formation and proliferation assays were performed to evaluate self-renewal ability of MSCs. Alcian blue staining and Sox9 expression were measured to evaluate chondrogenesis of MSCs. Secretion of pro-inflammatory cytokines IFN-γ, IL-2, IL-4 and IL-10, and expression of nuclear factor kappa B (NF-κB) subunit p65 were also assessed.

**Results:**

Hesperidin improved self-renewal ability and chondrogenesis of MSCs, inhibited secretion of pro-inflammatory cytokines IFN-γ, IL-2, IL-4 and IL-10, and suppressed the expression of p65. Overexpression of p65 was able to reverse the hesperidin inhibited secretions of pro-inflammatory cytokines, and abolish the enhancing effect of hesperidin on chondrogenesis of MSCs.

**Conclusion:**

Hesperidin could serve as a therapeutic agent to effectively enhance chondrogenesis of human MSCs by inhibiting inflammation to facilitate cartilage tissue repair.

**Electronic supplementary material:**

The online version of this article (10.1186/s12950-018-0190-y) contains supplementary material, which is available to authorized users.

## Background

Highly specialized articular cartilage consists of chondrocytes that are embedded in a rich extracellular matrix, mostly constituted by collagen, proteoglycans, and water [1, 2]. The cartilage provides a low friction on the articular surfaces of the bone, and serves as an impact absorber for joint movement by covering the load-bearing surface [3, 4]. It possesses a low capacity for repair and regeneration due to the absence of vascular, lymphatic and neural networks as well as the lack of sufficient progenitor cells within the cartilage [5, 6]. Hence, once articular cartilage is injured and damaged, it cannot be spontaneously repaired. In trauma or more commonly in degenerative joint diseases like rheumatoid arthritis and osteoarthritis, which affect at least 15% US population, structure and function of the articular cartilage tissues are often both impaired [7, 8]. Articular cartilage diseases undoubtedly bring a large financial burden to the individuals as well as the health system worldwide, and are therefore regarded as a foremost health problem particularly in developed countries [7, 9].

Tissue engineering provides an exciting alternative approach for treating articular cartilage diseases via the development of biological substitutes. Recent reports demonstrated that human stem cells, especially mesenchymal stem cells (MSCs), produced positive outcomes in the treatment of articular cartilage diseases [10]. Multiple approaches have been taken to utilize MSCs, with some efforts focusing on their in vitro expansion while others on in vitro differentiation. For instance, MSCs expanded in vitro were reported to be successfully transplanted into the defective articular cartilage of both animals and human patients, which underwent differentiation in vivo that eventually led to regeneration of osteochondral tissue [11]. In addition, human MSCs can be obtained from a variety of adult tissues, expanded with ease, and subsequently differentiated into matrix-producing chondrocytes in vitro [12, 13], eventually leading to the formation of hyaline articular cartilage. Due to the potential teratoma formation of pluripotent stem cells (e.g., induced pluripotent stem cells or embryonic stem cells), they are less preferable compared to MSCs in cartilage tissue engineering [14]. However, cartilage tissue engineering has yet to be proven effective in clinical use. The structural and functional properties of native articular cartilage have not been fully adopted by the tissue-engineered cartilage [15, 16]. Therefore, to date, there is no reliable long-term therapeutic strategy for articular cartilage repair [17]. Conventional therapies (e.g., microfracture, mosaicplasty and ACI) or traditional therapies (e.g., joint surgery) possess several shortcomings. In joint surgery, implantation of a prosthetic device is performed to replace the living cartilage tissue, which can marginally rescue joint functions but for merely 10–15 years. This procedure also poses additional risks of post-surgery complications, including infection and inflammation [18]. Thus, an efficient treatment to successfully repair or regenerate articular cartilage tissues is urgently needed.

Hesperidin is a natural flavonoid that possesses anti-inflammatory properties in many disease models. For example, hesperidin has been shown in rodent model to reduce inflammation as well as inflammatory pain through suppression of cytokine production, NF-κB activity, and oxidative stress [19]. In a similar manner, in a mouse model of skin damage induced by ultraviolet B irradiation, hesperidin was demonstrated to inhibit oxidative stress and inflammation [20], by down-regulation of cytokine production including TNF-α, IL-1β, IL-6 and IL-10 [21]. However, the effect of hesperidin on the immune responses during chondrogenesis of MSCs has not yet been reported.

In the current study, we hypothesized that hesperidin could enhance self-renewal and chondrogenesis of isolated human MSCs in vitro, which could then facilitate their in vitro expansion and differentiation at a large scale for clinical cartilage tissue repair.

## Results

### Hesperidin improves self-renewal ability of MSCs

**Fig. 1:**
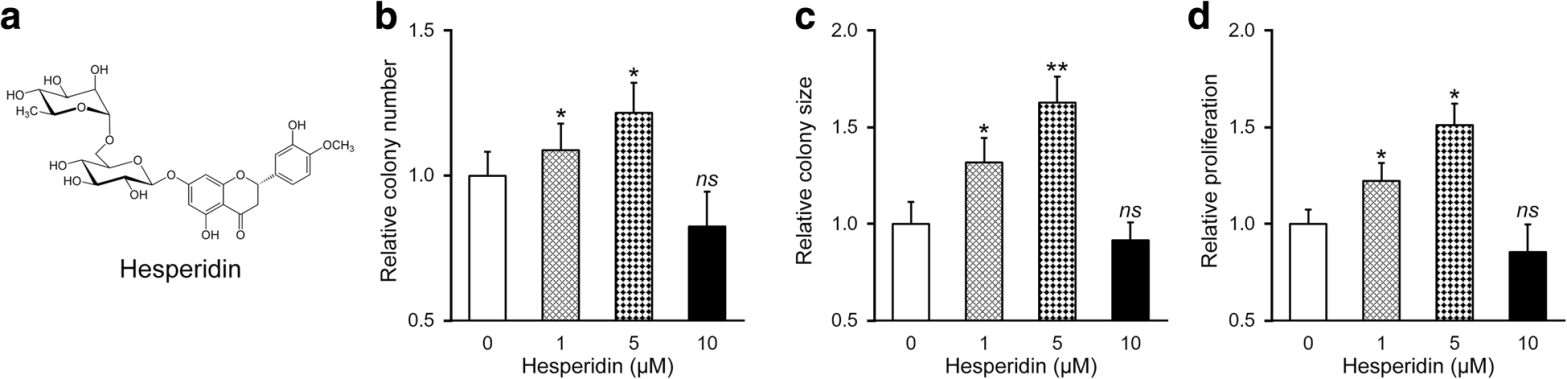
Hesperidin improves self-renewal ability of MSCs. **a** Chemical structure of hesperidin. **b** to **d** Colony number (**b**), colony size (**c**) and proliferation (**d**) of MSCs after treatments with 0, 1, 5 and 10 μM of hesperidin, respectively. Data were shown as mean ± SD from at least three independent experiments. * *p* < 0.05, ** *p* < 0.01, ns not significant, versus 0 μM hesperidin

The chemical structure of hesperidin was identified as shown in Fig. 1a. Here MSCs cells were challenged with different doses of hesperidin (0, 1, 5 and 10 μM), and the self-renewal capacity was assessed by colony formation and proliferation assays. Both the relative number and average size of colonies were significantly increased following hesperidin treatments up to 5 μM (Fig. 1b, c). Similarly, the cell viability determined using CCK-8 method clearly demonstrated that hesperidin markedly stimulated cell proliferation (Fig. 1d). However, high dose of hesperidin (10 μM in our system) induced slight inhibition on both colony formation and cell proliferation (Fig. 1b, c, d). Thus, 5 μM of hesperidin was chosen as the optimal dosage for the subsequent experiments in the current study, and to our best knowledge, these findings provided the first evidence that hesperidin improved self-renewal ability of patient-derived MSCs.

### Hesperidin enhances chondrogenesis of MSCs

**Fig. 2:**
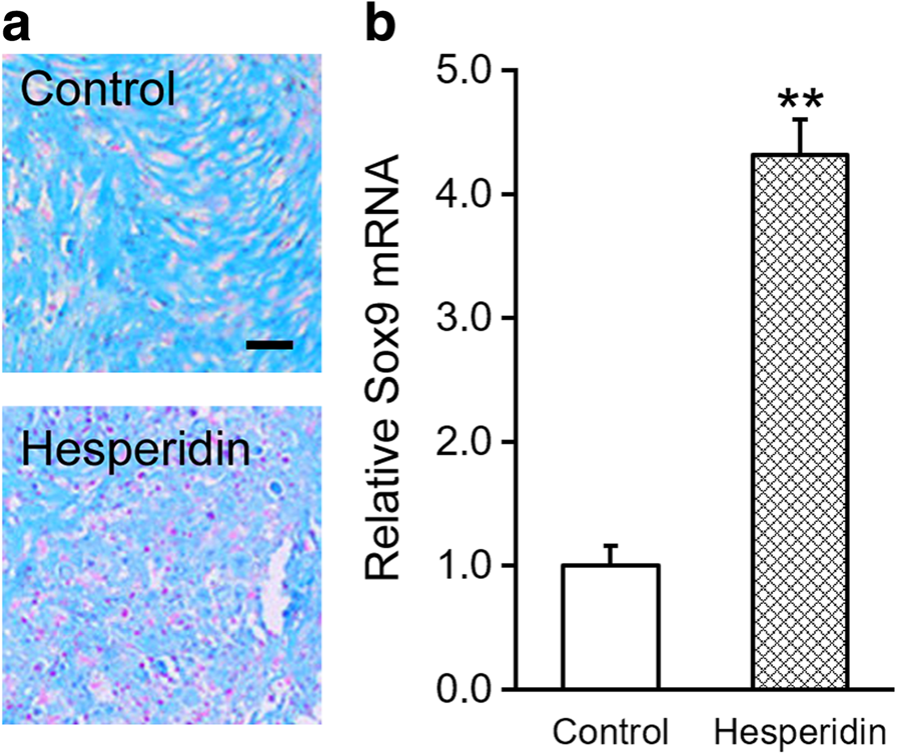
Hesperidin enhances chondrogenesis of MSCs. **a** At day 14 after differentiation induction in the absence (control) or presence of 5 μM hesperidin, the extents of chondrogenesis was evaluated by Alcian Blue staining assay. Images were representatives of at least three independent experiments, and positively stained cells were shown in pink, scale bar 100 μm. **b** At day 14 after differentiation induction in the absence (control) or presence of 5 μM hesperidin, the extents of chondrogenesis was evaluated by mRNA levels of chondrogenic marker Sox9. Data were shown as mean ± SD from at least three independent experiments. ** *p* < 0.01, versus control

Next, we sought to evaluate the possible effects of hesperidin on chondrogenesis potential of MSCs. Chondrogenesis was induced in the MSCs for 14 days upon hesperidin treatment. As shown in Fig. 2a, Alcian Blue staining showed significant increase of chondrogenesis in hesperidin-treated MSCs. These phenotypic observations were further confirmed at the molecular level by measuring specific chondrogenic marker Sox9, where hesperidin treatment induced evident up-regulation of Sox-9 (Fig. 2b). Our results clearly demonstrated that, besides self-renewal ability, hesperidin also enhanced chondrogenesis of MSCs.

### Hesperidin suppresses secretion of pro-inflammatory cytokines

**Fig. 3:**
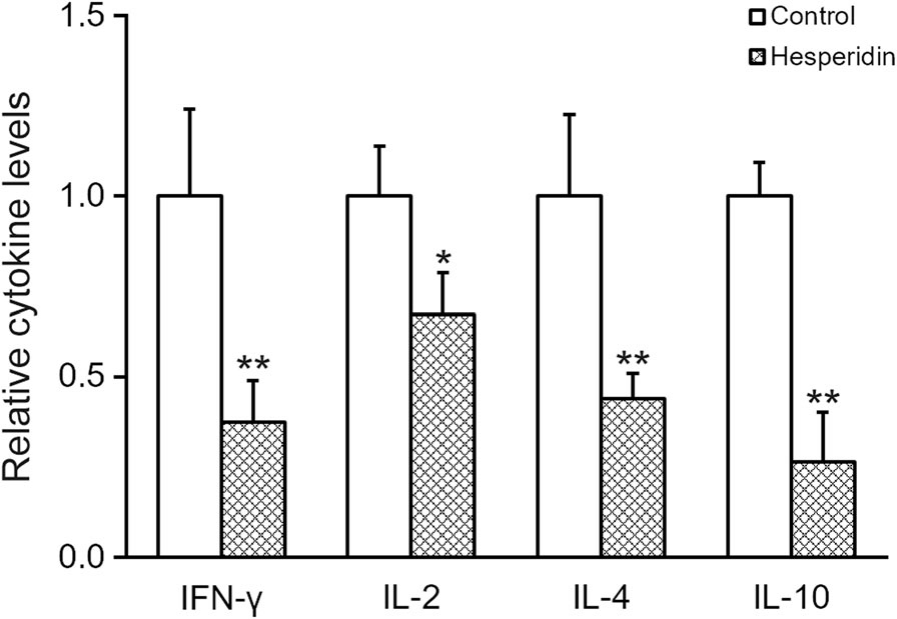
Hesperidin inhibits secretion of IFN-γ, IL-2, IL-4 and IL-10 of MSCs. MSCs were treated in the absence (control) or presence of 5 μM hesperidin, and levels of IFN-γ, IL-2, IL-4 and IL-10 in the medium were measured by ELISA. Data were shown as mean ± SD from at least three independent experiments. * *p* < 0.05, ** *p* < 0.01, versus control

Pro-inflammatory cytokines are essential players in both innate and acquired immune responses. Therefore, we subjected the MSCs in the absence or presence of 5 μM hesperidin, and then measured the levels of pro-inflammatory cytokines IFN-γ, IL-2, IL-4 and IL-10 in the medium using ELISA. Results clearly indicated that hesperidin treatment inhibited the secretion of all of abovementioned cytokines compared with those of control (Fig. 3).

### Hesperidin inhibits the expression of nuclear factor kappa B (NF-κB) subunit p65

**Fig. 4:**
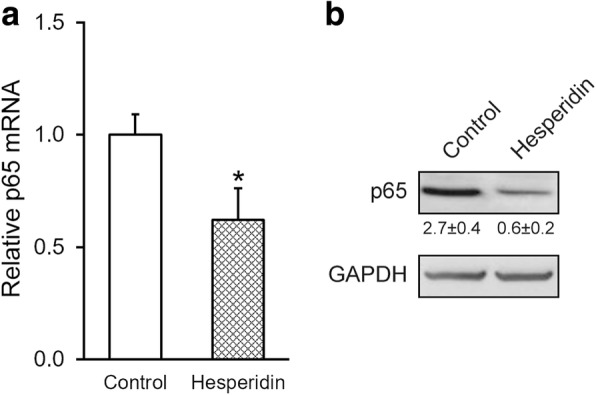
Hesperidin suppresses the expression of nuclear factor kappa B (NF-κB) subunit p65 of MSCs. MSCs were treated in the absence (control) or presence of 5 μM hesperidin, and relative mRNA (**a**) and protein (**b**) expressions of NF-κB subunit p65 were measured by RT-PCR and Western blot, respectively. Western blot was representative of at least three independent experiments, with relative intensity (p65/GAPDH) indicated below as mean ± SD. Data were shown as mean ± SD from at least three independent experiments. * *p* < 0.05, versus control

To determine the extent of inflammation, we examined the expression of biomarkers in inflammatory responses, such as NF-κB subunit p65. We treated the MSCs in the absence or presence of 5 μM hesperidin, and then examined the effect on expression of p65. We found that both mRNA and protein levels of p65 were significantly reduced by hesperidin treatment (Fig. 4a and b), indicating that hesperidin was able to inhibit the expression of NF-κB subunit p65.

Next, to confirm the effect of hesperidin were due to decreased p65 expression, we introduced p65 siRNA knockdown in the MSCs (Additional file 1: Figure S1A and S1B). As expected, p65 knockdown significantly inhibited secretions of pro-inflammatory cytokines IFN-γ, IL-2, IL-4 and IL-10 (Additional file 1: Figure S1C). The MSCs were then subjected to differentiation for 14 days, after which stronger extent of chondrogenesis was observed in p65 knockdown cells, in terms of Sox9 mRNA expression (Additional file 1: Figure S1D) and Alcian Blue staining (Additional file 1: Figure S1E).

### Inhibition of p65 is required for the inhibitory effect of hesperidin on cytokine secretions, and enhancing effect of hesperidin on chondrogenesis

**Fig. 5:**
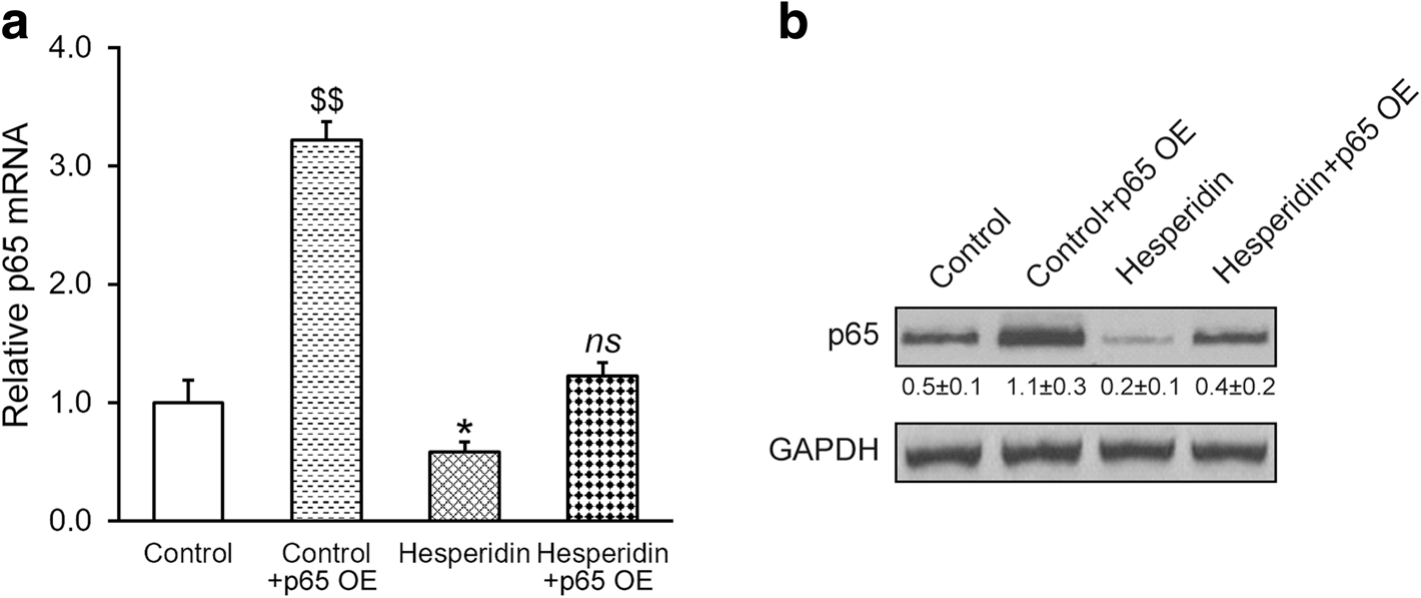
Effects of p65 overexpression (OE) in MSCs. Relative mRNA (**a**) and protein (**b**) expressions of NF-κB subunit p65 were measured by RT-PCR and Western blot, respectively, after the MSCs were transduced with either empty control or p65 lentiviral particle, in the absence (control) or presence of 5 μM hesperidin. Western blot was representative of at least three independent experiments, with relative intensity (p65/GAPDH) indicated below as mean ± SD. Data were shown as mean ± SD from at least three independent experiments. $$ *p* < 0.01, versus control, hesperidin and hesperidin+p65 OE. * p < 0.05, versus control and hesperidin+p65 OE. ns not significant, versus control

**Fig. 6:**
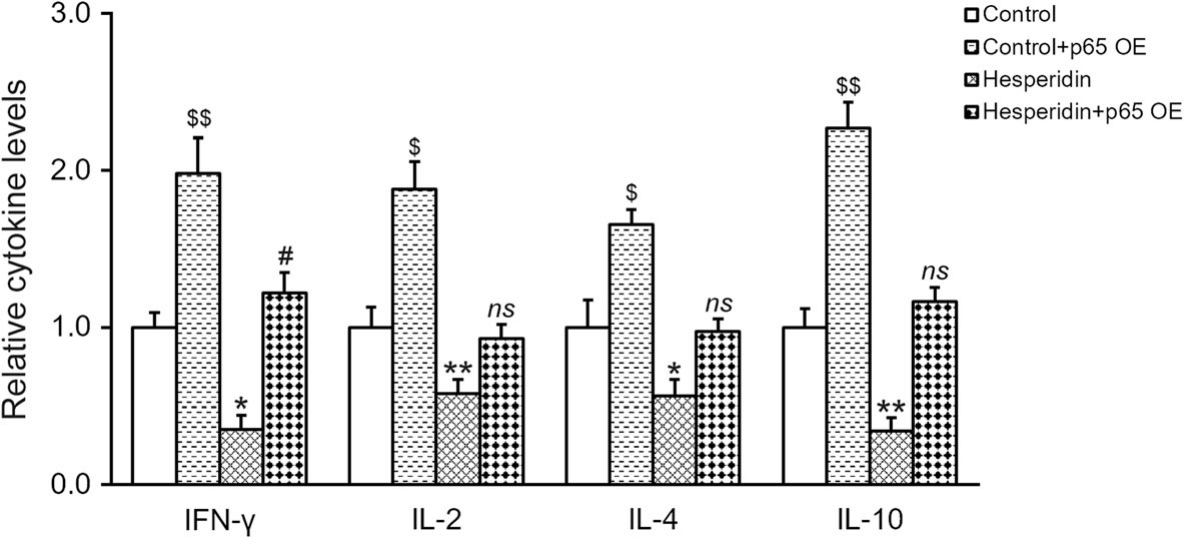
Inhibition of p65 is required for the inhibitory effect of hesperidin on IFN-γ, IL-2, IL-4 and IL-10 secretions from MSCs. MSCs were transduced with either empty control or p65 overexpression (OE) lentiviral particle, in the absence (control) or presence of 5 μM hesperidin, and levels of IFN-γ, IL-2, IL-4 and IL-10 in the medium were measured by ELISA. Data were shown as mean ± SD from at least three independent experiments. $$ p < 0.01, $ p < 0.05, versus control, hesperidin and hesperidin+p65 OE. * p < 0.05, ** p < 0.01, versus control and hesperidin+p65 OE. # p < 0.05, ns not significant, versus control

We then questioned whether the inhibitory effect of hesperidin on p65 contributed to the earlier observed suppression on cytokine secretions. To this end, we overexpressed p65 in MSCs, and verified that both mRNA and protein levels of p65 were greatly elevated compared to baseline in the absence (control) or presence of 5 μM hesperidin, respectively (Fig. 5a and b). Overexpression of p65 increased secretions of pro-inflammatory cytokines IFN-γ, IL-2, IL-4 and IL-10, which could be restored to baseline upon co-treatment of hesperidin (Fig. 6), suggesting that inhibition of p65 was indeed required for the inhibitory effect of hesperidin on cytokine secretions from MSCs.

**Fig. 7:**
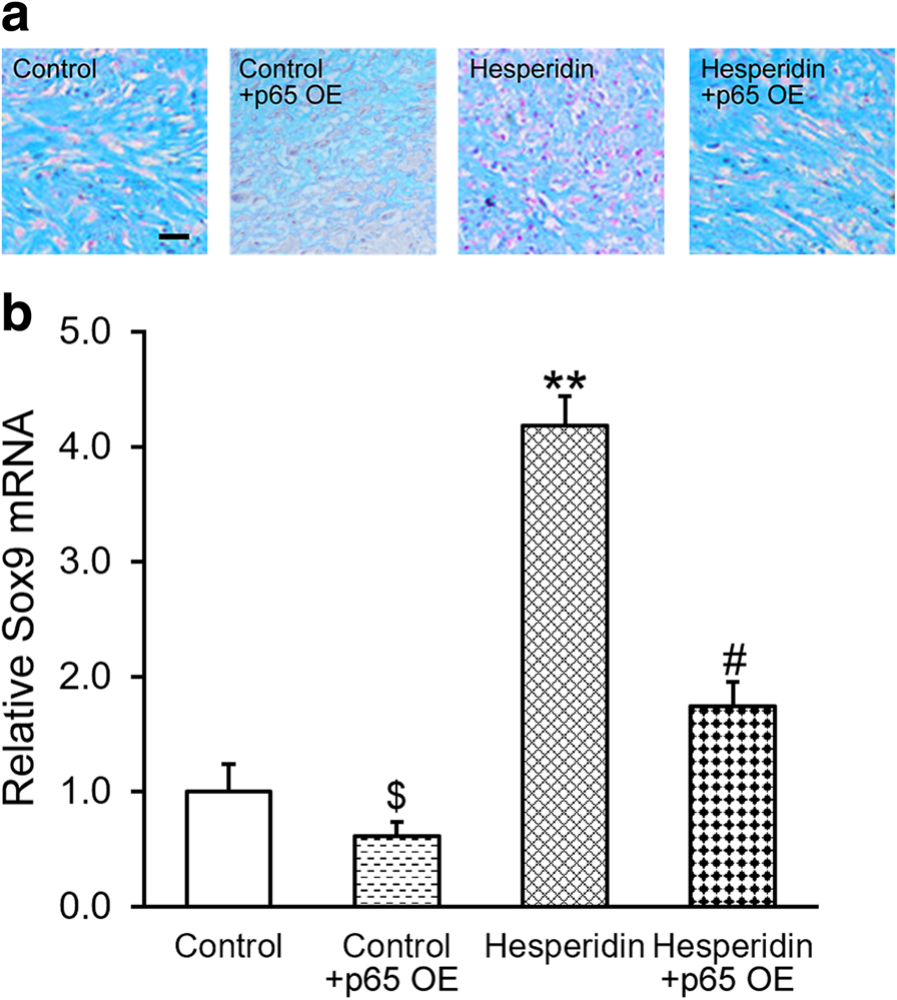
Inhibition of p65 is required for the enhancing effect of hesperidin on chondrogenesis of MSCs. MSCs were transduced with either empty control or p65 overexpression (OE) lentiviral particle, at day 14 after chondrogenesis induction in the absence (control) or presence of 5 μM hesperidin, the extents of chondrogenesis was evaluated by Alcian Blue staining assay (**a**), and by mRNA levels of chondrogenic marker Sox9 (**b**). Images were representatives of at least three independent experiments, and positively stained cells were shown in pink, scale bar 100 μm. Data were shown as mean ± SD from at least three independent experiments. $ p < 0.05, versus control, hesperidin and hesperidin+p65 OE. ** p < 0.01, versus control and hesperidin+p65 OE. # p < 0.05, versus control

Next, we further examined the effect of p65 inhibition on enhancing effect of hesperidin on chondrogenesis. MSCs were transduced with either empty control or p65 lentiviral particle, at day 14 after chondrogenesis induction in the absence (control) or presence of 5 μM hesperidin. Overexpression of p65 decreased chondrogenesis in terms of Alcian Blue staining (Fig. 7a) and Sox9 expression (Fig. 7b). Again, upon co-treatment of hesperidin, chondrogenesis extent was restored to baseline, indicating that inhibition of p65 was also required for the enhancing effect of hesperidin on chondrogenesis of MSCs.

## Discussion

In the study, we hereby reported for the first time that, hesperidin was able to improve self-renewal ability and chondrogenesis of MSCs, inhibit secretion of pro-inflammatory cytokines IFN-γ, IL-2, IL-4 and IL-10, and suppressed the expression of NF-κB subunit p65. Since human MSCs can be easily expanded and subsequently differentiated into matrix-producing chondrocytes [12, 13], eventually leading to the formation of hyaline articular cartilage, the observed enhancing effect of hesperidin on chondrogenesis of MSCs was potentially valuable in future clinical applications. In fact, hesperidin has been reported previously to exhibit beneficial effect toward cartilage tissues. In costal cartilage cells isolated from rabbits, a hesperidin loaded poly (lactic-co-glycolic acid) scaffold could improve attachment and proliferation of these cartilage cells, suggesting the potential of hesperidin in cartilage tissue engineering [22].

Furthermore, we overexpressed p65 in the context of hesperidin treatment, which reversed the hesperidin inhibited secretions of pro-inflammatory cytokines, and abolished the enhancing effect of hesperidin on chondrogenesis of MSCs. This result is intriguing in that it implicates the NF-κB signalling pathway and inflammation as the molecular mechanism underlying hesperidin action. NF-κB, a nuclear transcription factor, is shown to be involved in inflammatory, immune and stress responses. The signalling pathway of NF-κB include NF-κB, NF-κB inhibitor (IκB), IKKs upstream kinase and IκB kinase complex (IKKs). In mammals, NF-κB family is composed of NF-κB1, NF-κB2, p65/RelA, C-Rel and RelB, all of which contain a Rel homology domain (RHD) [23]. NF-κB forms heterodimer or homodimer in cells, and p65/NF-κB1 is the first discovered and most widely existing dimer. The conventional NF-κB signaling also predominantly involves the p65/NF-κB1 dimer [23]. NF-κB contributes to both innate and adaptive immune responses, and is one of the critical regulators of the production of pro-inflammatory cytokines [24]. The activation of NF-κB results in enhanced recruitment of inflammatory cells and increased production of pro-inflammatory mediators, including IL-1, IL-6, IL-8 and TNF. Inhibition of NF-κB activity has consistently proven effective in the control of inflammatory diseases in several animal models. For example, blockage of NF-κB activity suppressed both the inflammation and tissue damage in rheumatoid synovium [25]. There has been reports regarding the effects of hesperidin on NF-κB signal transduction using mouse models. In a diabetic mouse model, hesperidin reduced NF-κB level [26], whereas in a mouse model for pain hesperidin was shown to suppress the activity of NF-κB [19]. Further, hesperidin was also demonstrated to negatively regulate pro-inflammatory cytokines downstream of NF-κB, including IL-6 and IL-10 [21]. Our current study provides yet another instance supporting the anti-inflammatory properties of hesperidin, indicating that hesperidin may possess a universal anti-inflammatory function in various disease models, including but not limited to cartilage repair via tissue engineering using MSCs.

It is challenging to produce tissue-engineered cartilage resembling the native articular cartilage. Mechanical loading is regarded as a critical factor in cartilage tissue engineering for the reason that in daily activities normal articular cartilage is constantly subjected to mechanical loading [6, 27]. Mechanical loading is able to stimulate chondrogenesis in vitro and suppress hypertrophic differentiation of human MSCs [28, 29]. However, the particular type of mechanical loading and its loading regime to enhance non-hypertrophic chondrogenesis along with the mechano-transduction signalling remain to be clarified. This knowledge will help to establish a function-wise native-like tissue-engineered articular cartilage for therapeutic applications [27], and it would be of great clinical value to investigate whether hesperidin treatment could enhance the mechanical response of cartilage tissue derived from human MSCs.

Other factors contributing to successful cartilage tissue engineering using human MSCs include three-dimensional (3D) scaffold, and growth factors, both of which are reported to be essential for the quality of tissue-engineered cartilage [2, 10, 30]. In the context, the study by Cho et al. on the effect of hesperidin loaded poly (lactic-co-glycolic acid) scaffold costal cartilage cells [22] has demonstrated the usefulness of combining 3D scaffold with hesperidin. It will be interesting to further investigate the combinational effect of hesperidin with other contributing factors on MSC chondrogenesis in future studies.

## Conclusion

To conclusion, our current study demonstrates that hesperidin serves as a therapeutic agent to effectively enhance both self-renewal and chondrogenesis of human MSCs in vitro by inhibiting inflammation to facilitate cartilage tissue repair. Our current study therefore supports a wider application of hesperidin for multiple approaches to use MSCs for clinical cartilage tissue repair.

## Methods

### Human MSC culturing

The protocol for the use of human cells was approved by the committee of the Second Hospital of Shandong University, and bone marrow cells were harvested from bone fragments of patients from the Second Hospital of Shandong University, with written consent forms acquired from all patients. All bone marrow aspirates were diluted with low-glucose Dulbecco’s modified Eagle’s medium (DMEM, Thermo, Waltham, MA, USA), and then subjected to Ficoll gradient centrifugation (1200×g for 30 min at room temperature). The cells from the interface were harvested, followed by two washes in phosphate-buffered saline (PBS). Mononuclear cells re-suspended in complete DMEM were counted with a hemocytometer and seeded in 10 cm^2^ tissue culture dishes at a density of 5 × 10^6^ cells/10 mL. After two days, floating cells were discarded, and the adherent cells were kept for culture at 37 °C with 5% humidified CO_2_. After reaching a confluence of 75–85%, cells were detached with 0.05% trypsin/1 mM EDTA and re-plated, and expanded cells with < 9 passages were used in the experiments.

### Colony formation assay

A total of 1 × 10^5^ MSCs were plated into a 10-cm petri dish and continuously cultured for up to 21 days in the presence of 0, 1, 5 and 10 μM of hesperidin, respectively. Crystal violet (0.5%, SIGMA, MO, USA) was used to stain the formed colonies for 15 mins followed by counting under light microscope. Colonies larger than 2 mm in diameter were counted, which typically ranged from 50 to 200 per dish (calculated from 10 randomly chosen fields in each dish).

### Proliferation assay

The proliferation of cells was assessed by commercial CCK-8 kit (Dojindo, Kumamoto, Japan). In brief, 1 × 10^5^ MSCs were plated into each well of 6-well plate and continuously cultured for up to 7 days in the presence of 0, 1, 5 and 10 μM of hesperidin, respectively. 10 ul CCK-8 solution was then added into each well and the chromogenic reaction was carried out at 37 °C for 15 mins. Microplate reader (Molecular Devices, Sunnyvale, CA, USA) was used to record the absorption at 450 nm and relative cell viability was calculated.

### Chondrogenesis assays

MSCs were cultured in chondrogenic induction medium (DMEM, 0.2 mM ascorbate-2-phosphate, 20% FBS, and 10 mM glycerol-2-phosphate) for 14 days in the absence or presence of 5 μM hesperidin, with fresh medium exchanged every 2 days. Chondrogenesis was evaluated using Alcian Blue (Millipore, Billerica, MA, USA) staining.

### mRNA extraction and real-time PCR

Total mRNA was extracted using Trizol (Invitrogen, Carlsbad, CA, USA), and reverse transcribed to complementary cDNAs with Superscript II following manufacturer’s instructions (Biorad, Hercules, CA, USA). Triplicate PCR reactions were conducted using cyber green-based system (Applied Biosystems, Waltham, MA, USA) with the following conditions: 15 s at 95 °C, 1 min at 60 °C for 40 times. The relative expression levels were calculated using GAPDH as the internal control. Primers used in this study were: Sox9 forward 5′-GTA CCC GCA CTT GCA CAA-3′, reverse 5’-TCT CGC TCT CGT TCA GAA GTC-3′; p65 forward 5’-ACA TCC ATG CGG AGA ACG AGG AG-3′, reverse 5′-AGT GCT GCG AGT GAG TCA AGA GG-3′; GAPDH forward 5’-CTG ACT TCA ACA GCG ACA CC-3′, reverse 5′-TAG CCA AAT TCG TTG TCA TAC-3′.

### Western blot

Cell resuspension was prepared in the lysis buffer containing 150 mM NaCl, 50 mM Tris-HCl, 10 mM HEPES, 0.1% NP-40 alternative, 0.5 mM NaF, 0.25% Na-deoxycholate, 1 mM Na_3_VO_4_, pH 7.4 (Protease Inhibitor Cocktail, Roche, 1 tablet/10 ml). Cell lysates were quantitated using BCA protein assays, and 30 μg total protein was then run on SDS-PAGE followed by transfer to PVDF membranes. The membranes were subsequently blocked with 1% BSA (bovine serum albumin, Sigma, USA), and incubated with primary antibodies at 4 °C overnight. Primary antibodies for p65 and GAPDH were both purchased from Abcam. HRP conjugated secondary antibodies were utilized to visualize bands in an ECL-based imaging system.

### p65 overexpression and knockdown

Stable overexpression and knockdown of p65 were established using p65 lentiviral particle (LPP-F0160-Lv105) and p65 shRNA particle (HSH016213-CH1), both of which purchased from GeneCopoeia (Rockville, MD, USA). Cells were first transduced by respective lentiviral particles for 24 h, followed by selection with puromycin for 2 weeks, according to vendor’s instructions.

### Enzyme-linked immunosorbent assay (ELISA)

The MSCs were treated in the absence or presence of 5 μM hesperidin for 2 days. Cells were then completely removed by centrifugation and clear medium was collected for ELISA analysis. The levels of IFN-γ, IL-2, IL-4 and IL-10 were measured with the commercially available ELISA kits (Abcam, MA, USA) following the manufacturer’s instructions.

### Statistical analysis

All data were analyzed using SPSS 22.0 system (IBM, Armonk, NY, USA), and presented as mean ± standard deviation (SD) from at least three independent experiments. The differences between groups were determined by Student’s T tests and single factor variance analysis (ANOVA). *P* values less than 0.05 were considered statistical significant.

## Additional file

Additional file 1: **Figure S1.** p65 knockdown inhibits secretion of IFN-γ, IL-2, IL-4 and IL-10, and enhances chondrogenesis of MSCs. MSCs were transduced with control or siRNA against NF-κB subunit p65, followed by assessments of (A) mRNA and (B) protein expressions of p65, (C) levels of IFN-γ, IL-2, IL-4 and IL-10 in the medium. MSCs with either control or p65 siRNA were subjected to 14 days of differentiation induction, followed by assessments of (D) mRNA expression of chondrogenic marker Sox9, and (E) extents of chondrogenesis. Images were representatives of at least three independent experiments, and positively stained cells were shown in pink, scale bar 100 μm. Western blot was representative of at least three independent experiments, with relative intensity (p65/GAPDH) indicated below as mean ± SD. Data were shown as mean ± SD from at least three independent experiments. ** *p* < 0.01, * *p* < 0.05, versus control. (DOCX 519 kb)

## Abbreviations

MSCs: Mesenchymal stem cells
NF-κB: Nuclear factor kappa B
