# Supplementary material for: Anti-inflammatory effect of hesperidin enhances chondrogenesis of human mesenchymal stem cells for cartilage tissue repair
Source: J Inflamm (Lond). 2018 Jul 20;15:14. doi: 10.1186/s12950-018-0190-y (PMC6053785; doi:10.1186/s12950-018-0190-y)
Supplement: Supplementary file 1 — Figure S1. p65 knockdown inhibits secretion of IFN-γ, IL-2, IL-4 and IL-10, and enhances chondrogenesis of MSCs. MSCs were transduced with control or siRNA against NF-κB subunit p65, followed by assessments of (A) mRNA and (B) protein expressions of p65, (C) levels of IFN-γ, IL-2, IL-4 and IL-10 in the medium. MSCs with either control or p65 siRNA were subjected to 14 days of differentiation induction, followed by assessments of (D) mRNA expression of chondrogenic marker Sox9, and (E) extents of chondrogenesis. Images were representatives of at least three independent experiments, and positively stained cells were shown in pink, scale bar 100 μm. Western blot was representative of at least three independent experiments, with relative intensity (p65/GAPDH) indicated below as mean ± SD. Data were shown as mean ± SD from at least three independent experiments. ** p < 0.01, * p < 0.05, versus control. (DOCX 519 kb) [file 12950_2018_190_MOESM1_ESM.docx]

**
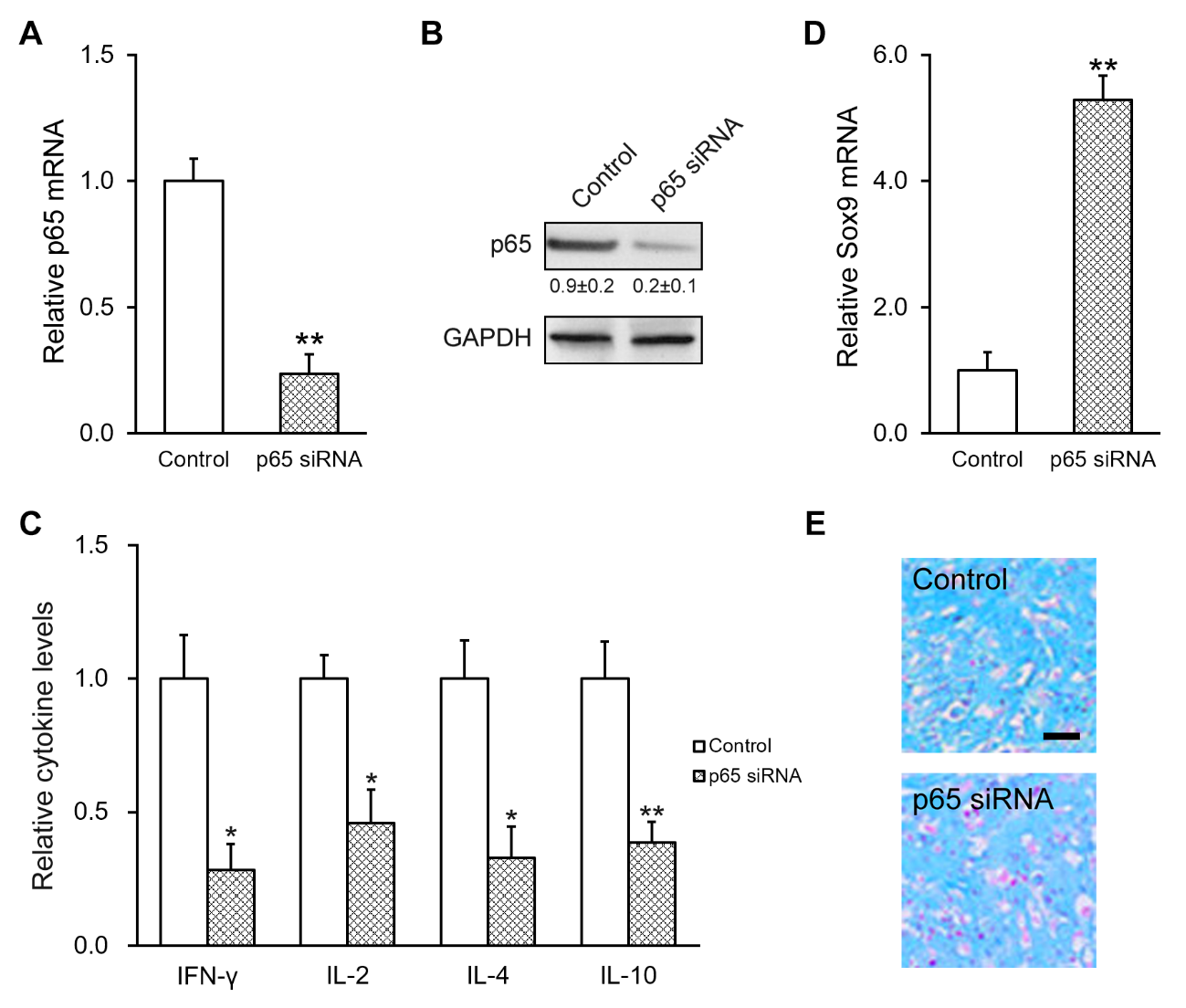
**Additional file 1**: Figure S1. p65 knockdown** **inhibits secretion of IFN-γ, IL-2, IL-4 and IL-10, and enhances chondrogenesis of MSCs.** MSCs were transduced with control or siRNA against NF-κB subunit p65, followed by assessments of (A) mRNA and (B) protein expressions of p65, (C) levels of IFN-γ, IL-2, IL-4 and IL-10 in the medium. MSCs with either control or p65 siRNA were subjected to 14 days of differentiation induction, followed by assessments of (D) mRNA expression of chondrogenic marker Sox9, and (E) extents of chondrogenesis. Images were representatives of at least three independent experiments, and positively stained cells were shown in pink, scale bar 100 μm. Western blot was representative of at least three independent experiments, with relative intensity (p65/GAPDH) indicated below as mean ± SD. Data were shown as mean ± SD from at least three independent experiments. ** p < 0.01, * p < 0.05, versus control.
